# PPARγ Ligands Switched High Fat Diet-Induced Macrophage M2b Polarization toward M2a Thereby Improving Intestinal *Candida* Elimination

**DOI:** 10.1371/journal.pone.0012828

**Published:** 2010-09-20

**Authors:** Lise Lefèvre, Amandine Galès, David Olagnier, José Bernad, Laurence Perez, Rémy Burcelin, Alexis Valentin, Johan Auwerx, Bernard Pipy, Agnès Coste

**Affiliations:** 1 UMR-MD3 EA2405 Polarisation des Macrophages et Récepteurs Nucléaires dans les Pathologies Inflammatoires et Infectieuses, Université de Toulouse III, Toulouse, France; 2 UMR-MD3, RH2PT Université de la Méditerranée - Ministère de la Défense, Marseille, France; 3 Rangueil Institute of Molecular Medicine, I(2)MR, U858 Inserm, IFR31,Toulouse, France; 4 Laboratory of Integrative and Systems Physiology (LISP), Ecole Polytechnique Fédérale de Lausanne, Lausanne, Switzerland; University of Birmingham, United Kingdom

## Abstract

Obesity is associated with a chronic low-grade inflammation that predisposes to insulin resistance and the development of type 2 diabetes. In this metabolic context, gastrointestinal (GI) candidiasis is common. We recently demonstrated that the PPARγ ligand rosiglitazone promotes the clearance of *Candida albicans* through the activation of alternative M2 macrophage polarization. Here, we evaluated the impact of high fat diet (HFD)-induced obesity and the effect of rosiglitazone (PPARγ ligand) or WY14643 (PPARα ligand) both on the phenotypic M1/M2 polarization of peritoneal and cecal tissue macrophages and on the outcome of GI candidiasis. We demonstrated that the peritoneal macrophages and the cell types present in the cecal tissue from HF fed mice present a M2b polarization (TNF-α^high^, IL-10^high^, MR, Dectin-1). Interestingly, rosiglitazone induces a phenotypic M2b-to-M2a (TNF-α^low^, IL-10^low^, MR^high^, Dectin-1^high^) switch of peritoneal macrophages and of the cells present in the cecal tissue. The incapacity of WY14643 to switch this polarization toward M2a state, strongly suggests the specific involvement of PPARγ in this mechanism. We showed that in insulin resistant mice, M2b polarization of macrophages present on the site of infection is associated with an increased susceptibility to GI candidiasis, whereas M2a polarization after rosiglitazone treatment favours the GI fungal elimination independently of reduced blood glucose. In conclusion, our data demonstrate a dual benefit of PPARγ ligands because they promote mucosal defence mechanisms against GI candidiasis through M2a macrophage polarization while regulating blood glucose level.

## Introduction

Obesity is associated with a chronic low-grade inflammation that predisposes to insulin resistance and development of type 2 diabetes. Increased adiposity promotes macrophage infiltration into adipose tissue, leading to a local inflammation and insulin resistance. Adipose tissue macrophages (ATMs) consist of at least two different phenotypes, i.e, classically activated pro-inflammatory M1 macrophages and alternatively activated M2 macrophages. Indeed, a recent study suggests that diet-induced obesity induces the recruitment of M1 pro-inflammatory polarized macrophages in adipose tissue participating in a state of insulin resistance [1]. In addition, another study also demonstrates that mice deleted for PPARγ, a nuclear receptor involved in adipocyte differentiation and macrophage M2 alternative polarization [2], displayed insulin resistance with reduced number and impaired function of M2 macrophages [3]. Conversely, a recent report proposed that the chronic inflammatory alterations during fat mass development are associated with increased abundance of macrophages in adipose tissue which present a particular M2 remodelling phenotype. These macrophages resembled M2 macrophages phenotypically by surface expression of Mannose Receptor (MR), CD163 and integrin avb5, their endocytic activity and production of anti-inflammatory cytokines (IL-10, IL-1Ra), but represent an unique type of macrophages that also secrete large amounts of pro-inflammatory cytokines. Moreover, they showed an endocytic activity similar to M2 macrophages and accordingly secreted high amounts of IL-10 and IL-1 receptor antagonist. However, basal and induced secretion of pro-inflammatory mediators TNF-α, IL-6, IL-1β, MCP-1 and MIP-1α were even higher in ATMs than in proinflammatory M1 macrophages [4]. This macrophage phenotype is similar to the M2b macrophages established by Mantovani *et al*, characterized by abundant levels of non-opsonic receptors (e.g. the MR, CD36) and high levels of inflammatory cytokine production with concomitant high IL-10 and low IL-12 [5], [6]. These authors have also highlighted other 2 subtypes of M2 macrophages, M2a and M2c, characterized by abundant levels of non-opsonic receptors and by low production of proinflammatory cytokines (IL-1β, TNF-α and IL-6). Although several studies have focused on the phenotype of ATM during metabolic dysregulation, no study has explored the M1/M2 polarization of macrophages in other tissues during metabolic deregulation.

A causal association between obesity and type II diabetes and an increased susceptibility to digestive infectious agents is well recognised [7]–[10]. Among these infections, *Candida* species have been frequently isolated from the oral cavities and GI tract of patients with diabetes mellitus [11]. A large number of reports suggest that *C. albicans* is the most common species identified in the oral and GI mucosa of these sensitive patients. The predisposition of the diabetics to infections by pathogenic fungal species has been explained in terms of enhancement of yeast growth by elevated tissue fluid glucose levels. Moreover, the presence of a high concentration of salivary glucose combined with low salivary secretion may enhance growth of yeasts and their adherence in epithelial oral cells [12]. Thus, as the macrophages are key cells in fungal elimination, it is important to determinate their phenotype in digestive mucosal tissues.

We previously reported that IL-13, a Th2 cytokine, promotes *in vitro* and *in vivo* the elimination of *C. albicans* by increasing the expression at the surface of macrophages of MR and Dectin-1, C-type lectin receptors involved in non-opsonized *C.albicans* recognition and phagocytosis and also in the production of reactive oxygen species [13]–[15]. In addition, we demonstrated that this increase of MR and Dectin-1 expression by IL-13, characteristic of M2 polarization, involved the activation of the nuclear receptor PPARγ. We also showed that this macrophage M2 activation by IL-13 can also be reproduced by rosiglitazone, a synthetic PPARγ ligand used as an antidiabetic drug. Conversely, we determined that IFNγ, a Th1 cytokine, inhibits the non-opsonized *C.albicans* phagocytosis by decreasing C-type lectin receptor expression on macrophage surface, but eliminates yeast by increasing the expression of opsonized receptors at the surface of macrophages and the macrophage inflammatory properties [16]. All these data clearly demonstrate the complementarities of these 2 types of macrophage polarization in the elimination of fungal infection.

In the present study, we evaluated the impact of insulin resistance induced by high fat diet (HFD) and the effect of rosiglitazone (PPARγ ligand) or WY14643 (PPARα ligand) on the phenotypic differentiation of peritoneal macrophages and of the cell types present in the cecal tissue. We then determined whether their polarization could modify their anti-fungal functions. We demonstrated for the first time that the peritoneal macrophages and the cell types present in the cecal tissue from HF fed mice present a M2b polarization. Interestingly, only rosiglitazone treatment induces a phenotypic switch of macrophages into M2a state. In addition, we show that the M2b macrophage polarization in insulin resistant mice is associated with an increased susceptibility to GI fungal infections, whereas M2a polarization after rosiglitazone treatment favours the GI candidiasis elimination independently of reduced blood glucose. These findings demonstrate in a hyperglycaemic metabolic context a dual benefit of PPARγ ligands, which are able to regulate blood glucose level and strengthen M2a defence of macrophages during fungal infections.

## Results

### Validation of diet-induced diabesity mice model

**Figure 1:**
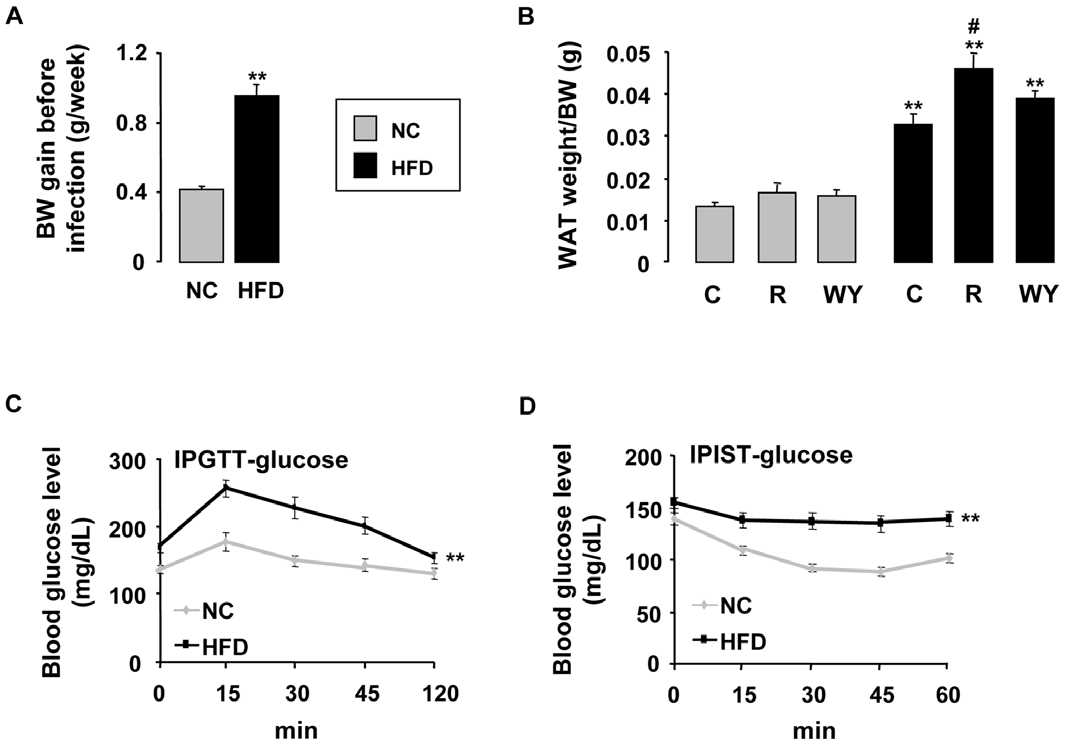
Validation of diet-induced diabesity mice model. (A) Weight gain of C57BL/6 mice (n = 18) after three months of chow (NC) or high fat (HF) diet. (B) Weight of the epididymal white adipose tissue measured after euthanasia of chow or HF fed mice treated or not with rosiglitazone (R) or WY 14643 (WY) (28 µg/10 g of mouse) (n = 6). (C) Serum glucose levels evolution during an i.p. glucose tolerance test performed after a glucose load (1 g/kg) in chow or HF fed, 6 hrs-fasted mice. The asterisks refer to all time points. (D) Serum glucose levels evolution during an i.p. insulin sensitivity test performed after an insulin load (0.5 U/kg) in chow or HF fed, 6 hrs-fasted mice. The asterisks refer to all time points. Data are represented as mean ± SEM **p<0.01 compared to NC control (NC C). #p<0.05 compared to HF control (HF C). The data are representative of three independent experiments.

We compared weight evolution of mice fed either chow or HFD for three months (Figure 1A). When fed a HFD, mice gained significantly more weight than mice fed standard chow. The differences in body weight were accounted for by an increased fat mass, as mirrored by the weight of the epididymal white adipose tissue (Figure 1B). In addition, WY 14643 treatment do not change the weight of white adipose tissue of mice both under chow and HF fed conditions. Rosiglitazone increases the weight of white adipose tissue of mice under HF diet, confirming the role of this drug in adipocyte differentiation.

The increase in body weight in mice under HFD is accompanied by a reduced metabolic control as evidenced by the higher fasting plasma glucose. Furthermore, mice in HF fed condition cleared glucose less effectively after i.p. glucose injection than animals fed chow diet (Figure 1C). The rate of glucose clearance upon i.p. insulin injection was also lower in mice fed HFD, further suggesting insulin resistance (Figure 1D). Altogether these data demonstrate that after three months of HFD mice developed all the characteristics of type 2 diabetes.

### Macrophage M2 polarization induced by HFD is potentiated by rosiglitazone

**Figure 2:**
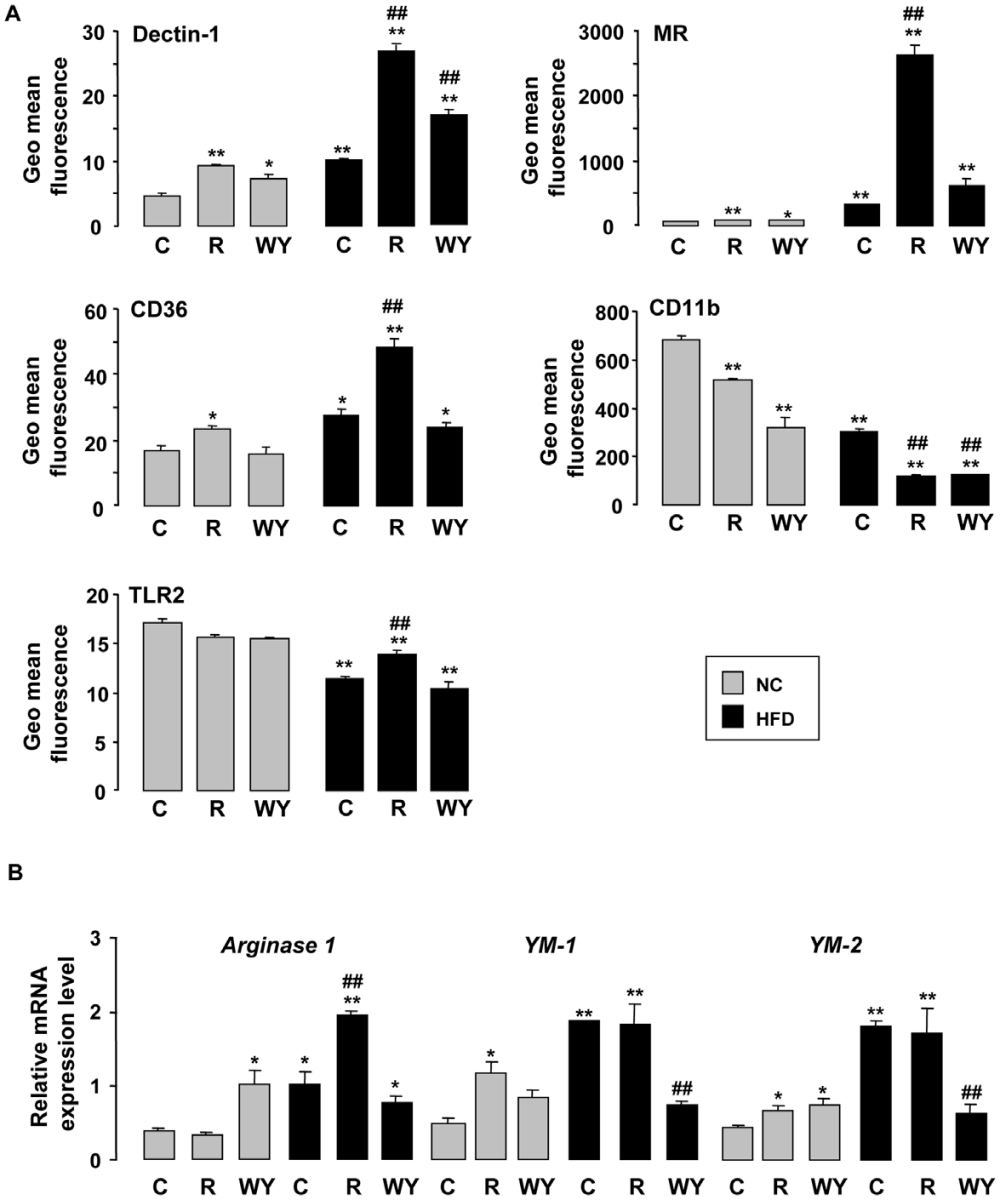
HFD-induced peritoneal macrophage M2 polarization and rosiglitazone treatment potentiated this phenotype. (A) Dectin-1, MR, CD36, CD11b and TLR2 protein expressions on peritoneal macrophages from mice fed a chow (NC) or a HFD (HF) and treated or not (C) with rosiglitazone (R) or WY14643 (WY) evaluated by flow cytometry (n = 6). (B) *Arginase 1*, *YM-1* and *YM-2* mRNA expressions on peritoneal macrophages from mice fed a chow (NC) or a HFD (HF) and treated or not (C) with rosiglitazone (R) or WY14643 (WY) by RT-PCR (n = 6). Data are represented as mean ± SEM *p<0.05 and **p<0.01 compared to NC control (NC C); #p<0.05 and ##p<0.01 compared to HF control (HF C). The data are representative of three independent experiments.

To define the polarization of peritoneal macrophages from untreated or treated mice in chow and HF fed conditions, the expression of M1 and M2 macrophage activation state markers was assessed by flow cytometry and RT-PCR (Figure 2). We observed that the peritoneal macrophages from mice in HF fed condition express more strongly the proteins encoding for the MR, Dectin-1 and CD36, established markers of alternative M2 macrophage activation (Figure 2A). In addition, *YM-1*, *YM-2* and *Arginase 1* mRNA levels, other M2 markers, are also increased by HFD (Figure 2B). In contrast, HFD decreases CD11b expression, a M1 polarization marker, at the surface of macrophages. Interestingly, following rosiglitazone treatment, the expression of the MR, Dectin-1, CD36 and TLR-2, receptors known to be involved in pathogen elimination, at the surface of macrophages from mice both in chow and HF fed conditions were significantly higher (Figure 2A). In contrast, the rosiglitazone treatment decreased CD11b expression. Moreover, the treatment with WY14643 increases slightly Dectin-1 expression at the macrophage surface and does not alter the expression of other receptors involved in innate immune defence. As rosiglitazone, WY14643 treatment decreased CD11b expression. These data demonstrate that HFD favours the polarization of peritoneal macrophages towards a M2 profile and that this differentiation is strongly potentiated by rosiglitazone, whereas WY14643 has a weaker effect.

### Macrophage M2b polarization induced by HFD is switched by rosiglitazone in an M2a state

**Figure 3:**
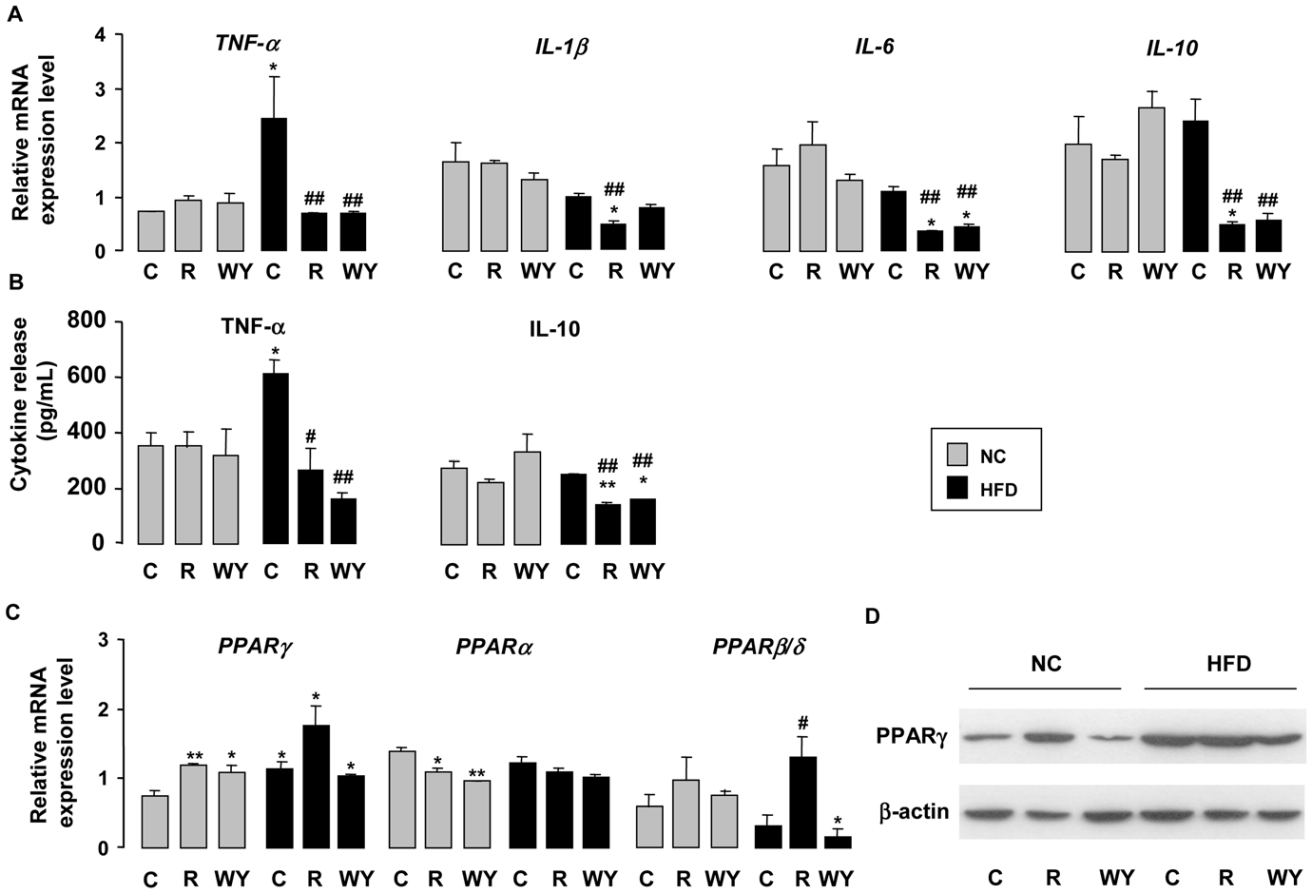
Rosiglitazone switches HFD-induced M2b polarization towards M2a. *(A) TNF-α*, *IL-1β*, *IL-6 and IL-10* mRNA expressions in peritoneal macrophages from mice fed a chow (NC) or a HFD (HF) and treated or not (C) with rosiglitazone (R) or WY14643 (WY) by RT-PCR (n = 6). (B) TNF-α and IL-10 release by peritoneal macrophages from mice fed a chow (NC) or a HFD (HF) and treated or not (C) with rosiglitazone (R) or WY14643 (WY) by ELISA (n = 6). (C) *PPARγ*, *PPARα* and *PPARβ/δ* mRNA expressions in peritoneal macrophages from mice fed a chow (NC) or a HFD (HF) and treated or not (C) with rosiglitazone (R) or WY14643 (WY) by RT-PCR (n = 6). (D) PPARγ protein level in peritoneal macrophages from mice fed a chow (NC) or a HFD (HF) and treated or not (C) with rosiglitazone (R) or WY14643 (WY) by western blot. Data are represented as mean ± SEM *p<0.05 and** p<0.01 compared to NC control (NC C); #p<0.05 compared to HF control (HF C). The data are representative of three independent experiments.

To establish the precise phenotype of peritoneal macrophages from untreated or treated mice in chow and HF fed conditions, we evaluated the transcripts for some inflammatory cytokines (Figure 3A). In parallel, the capacity of these macrophages to produce TNF-α and IL-10 cytokines in response to *C. albicans* was also tested (Figure 3B). *TNF-α* mRNA levels increased significantly in macrophages from mice under HFD. This finding was further reflected by the increase of TNF-α production by these macrophages in response to *Candida* challenge (Figure 3B). The expression of *IL-6*, *IL-1β* and *IL-10* mRNA is not affected by HFD.

Furthermore, rosiglitazone and WY14643 treatments of mice under HFD conditions induced a decrease in the expression of *TNF-α*, as well as in that of two other pro-inflammatory cytokines (*IL-6* and *IL-1β*). The anti-inflammatory cytokine *IL-10* mRNA level in macrophages from mice under HFD is also reduced by the treatments with rosiglitazone and WY14643 (Figure 3A). In line, the same profile of IL-10 production by macrophages in response to fungal challenge was obtained (Figure 3B). Overall, these results demonstrate that HFD induced a M2b (TNFα^high^, IL-10^high^, MR, Dectin-1) polarization and rosiglitazone switches the polarization from an M2b to an M2a (TNFα^low^, IL-10^low^, MR^high^, Dectin-1^high^) phenotype.

### HFD-induced M2b polarization and rosiglitazone-induced M2a polarization are associated with an up-regulation of PPARγ expression

Because PPARγ regulates the MR, Dectin-1 and CD36 [13], [15], [17], characteristic markers of M2 macrophage, we evaluated PPARγ mRNA and protein levels in macrophages (Figure 3C and D). PPARγ mRNA and protein levels are significantly increased in macrophages from mice under HFD (Figure 3C and D). The expression of *PPARα* mRNA is not modified by the HFD and by rosiglitazone (Figure 3C). Under HFD conditions *PPARδ/β* is increased by rosiglitazone treatment, confirming that *PPARδ/β* is induced by rosiglitazone *via* a PPARγ dependent mechanism [18] (Figure 3C). These data demonstrate that the specific increase of PPARγ mRNA and protein levels by HFD is correlated with the orientation towards M2 macrophage phenotype. In this state, the macrophages are specifically sensitive to rosiglitazone PPARγ ligand treatment.

### HFD and rosiglitazone trigger the recruitment to the cecal tissue of M2 macrophages

**Figure 4:**
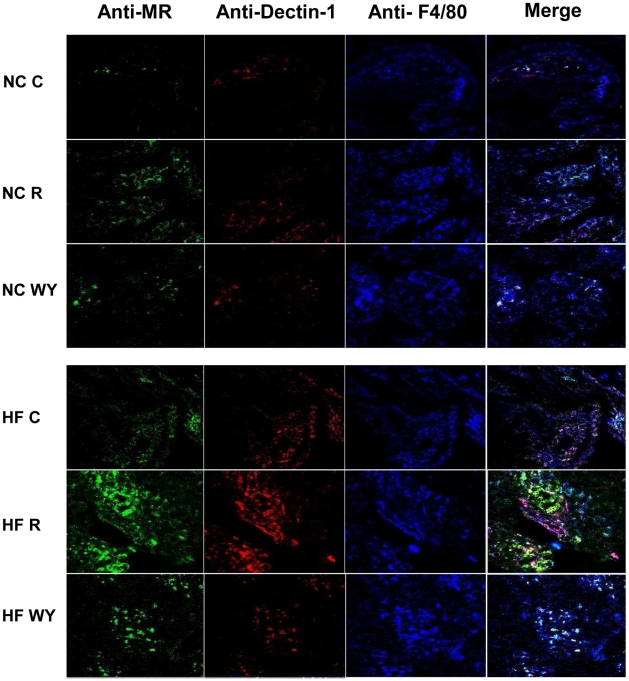
HFD and rosiglitazone treatment trigger recruitment to the cecal tissue of M2 macrophages that over express the MR and Dectin-1. Confocal laser microscopy of cecum tissues from untreated (C), rosiglitazone (R) or WY14643 treated chow (NC) or HF fed mice. The blue color represents the macrophages; red represents Dectin-1 positive cells; green represents MR positive cells. Merged picture of green, red and blue colors represents triple positive cells. Staining is representative of at least three different cecal tissue samples of three animals by group.

As diabetes mellitus is associated with infectious and inflammatory disorders of GI tract, we determine the recruitment and the M1/M2 phenotype of macrophages present in the cecal tissue by confocal microscopy. As expected, an increase in the recruitment of macrophages in the cecal tissue was observed upon feeding a HFD (HF C) (Figure 4). Some macrophages are positive for both the MR and Dectin-1, strongly suggesting a M2 polarization. Interestingly, following rosiglitazone treatment a significantly larger number of macrophages are both MR and Dectin-1 positive, demonstrating that the cecal macrophages are mainly polarized M2 (NC R and HF R). These results demonstrate that the HFD promotes the recruitment of M2 macrophages to the cecal tissue and that rosiglitazone amplifies this infiltration.

**Figure 5:**
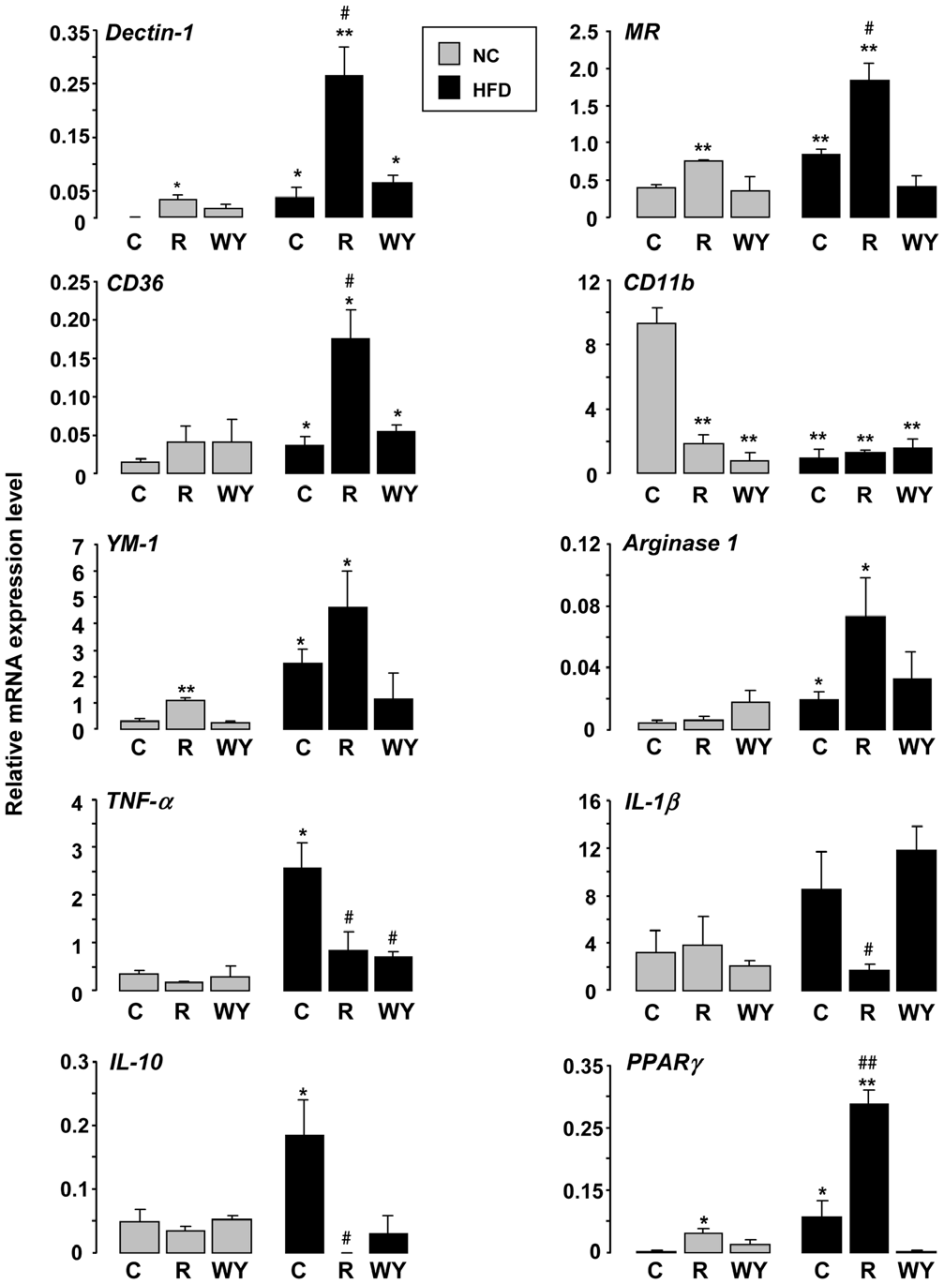
The cell types present in the cecal tissue from HFD mice exhibit a M2b phenotype which is shifted by rosiglitazone towards M2a. *Dectin-1*, *MR*, *CD36*, *CD11b*, *YM-1*, *Arginase 1*, *TNF-α*, *IL-1β*, IL-10 and *PPARγ mRNA* expressions on cecal tissue from mice fed a chow (NC) or a HFD (HF) and treated or not (C) with rosiglitazone (R) or WY14643 (WY) evaluated by RT-PCR (n = 6). Data are represented as mean ± SEM *p<0.05 and **p<0.01 compared to NC control (NC C); #p<0.05 compared to HF control (HF C). The data are representative of three independent experiments.

In the digestive cecal tissue, we have evaluated the mRNA levels of macrophage specific markers and the inflammatory profile of this tissue by RT-PCR. We showed that *Dectin-1*, *MR*, *CD36*, *YM-1 and Arginase 1* mRNA levels, established M2 markers, are increased in cecal tissue from mice in HF fed conditions (Figure 5). However, HFD decreases *CD11b* expression, a M1 polarization marker. The expressions of the *Dectin-1*, *MR*, *CD36*, *YM-1 and Arginase 1* in cecal tissue from mice under chow diet were significantly higher after rosiglitazone treatment. These increased expressions were potentiated in cecal tissue from mice under HFD. Moreover, the treatment with WY14643 did not change the mRNA profile of cecal tissue from mice under HFD. In addition, the cytokine profile showed an increase of proinflammatory cytokines (*TNF-α*, IL-1β) and also of *IL-10* anti-inflammatory cytokine in cecal tissue from mice under HF diet. In addition, rosiglitazone and WY14643 treatments of mice under HFD conditions induced a decrease in the expression of *TNF-α and IL-10*. The similarity between the mRNA profiles obtained in peritoneal macrophages and in the cecal tissue suggests that mucosal macrophages from HFD mice exhibit a M2b phenotype and that rosiglitazone shifts this M2b polarization towards a M2a phenotype. As in peritoneal macrophages, *PPARγ* mRNA level is increased in digestive cecal tissue from mice under HFD and rosiglitazone treatment potentiates this increase, confirming the involvement of PPARγ in the orientation towards M2 phenotype.

### Rosiglitazone, but not WY 14643, decreases *Candida* GI colonization in insulin resistant mice

**Figure 6:**
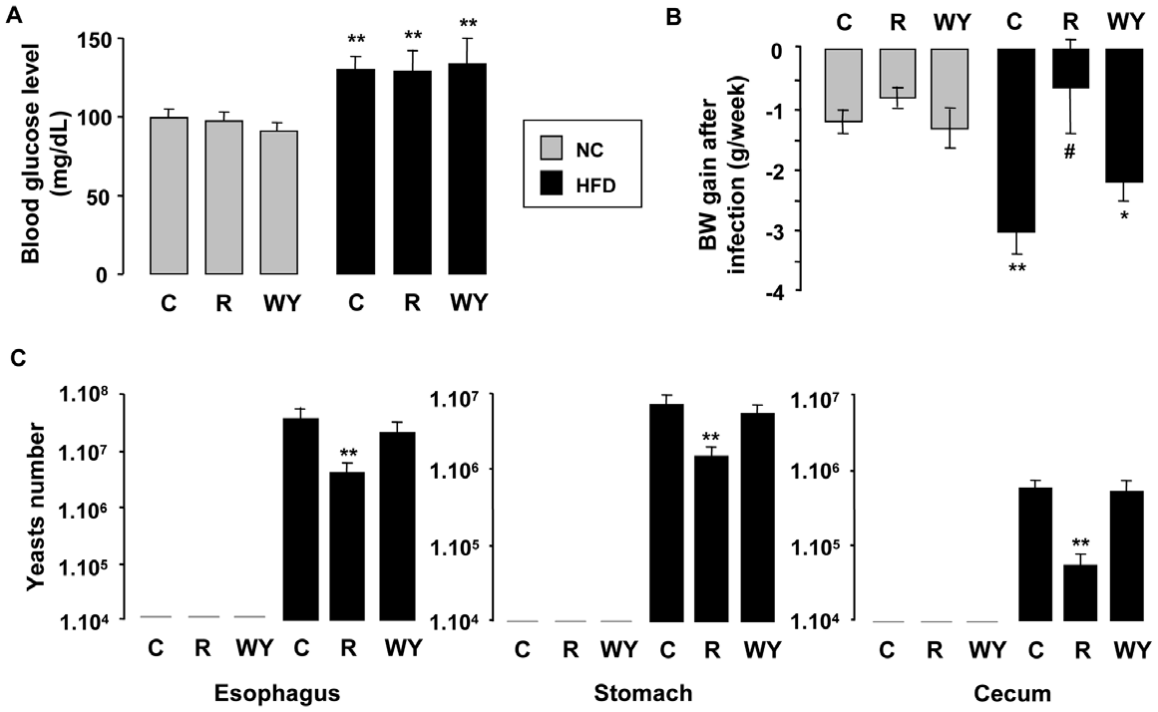
Rosiglitazone attenuates GI candidiasis during type II diabetes. (A) Blood glucose levels of mice fed a chow (NC) or a HFD after infection with *C. albicans* (50.10^6^ yeasts/mouse) and treatments with rosiglitazone (R) and WY14643 (WY) (n = 6). (B) Weight gain of these mice after infection with *C. albicans*. (C) *C. albicans* GI colonization of esophagus, stomach and cecum of mice fed a HFD and treated or not (HF C) with rosiglitazone (HF R) and WY14643 (HF WY) evaluated by RT-PCR. Data are represented as mean ± SEM *p<0.05 and **p<0.01 compared to NC control (NC C); #p<0.05 compared to HF control (HF C). The data are representative of three independent experiments.

To evaluate the impact of digestive mucosal macrophages polarization induced by HFD and rosiglitazone or WY14643 treatments on the outcome of gastrointestinal candidiasis, common infection during metabolic disorders, we subjected diet-induced diabesity mice to oral inoculation with *C. albicans*. After infection, the mice under HFD have a high fasting blood glucose level and short term rosiglitazone or WY14643 treatments did not affect blood glucose level (Figure 6A).

After one week of infection with *C.albicans*, the body weight of mice, which reflects their general health condition, was followed (Figure 6B). The mice under HFD loose more weight than mice under chow diet. When fed a chow diet, the mice have a slight loss of weight due to the gavage. Moreover, only rosiglitazone treatment of mice under HFD reverses significantly this weight loss. These data are in line with the evaluation of *C.albicans* GI colonization (Figure 6C). Indeed, in this model of candidiasis, the yeast only extensively colonized the oesophagus and the GI tract of mice under HF diet. No yeast was detectable in GI tract of mice fed chow diet (<10^4^), demonstrating that these lean mice are able to resolve the infection and that this metabolic dysregulation favors *Candida* infection. Interestingly, after treatment with rosiglitazone the number of yeast in oesophagus, stomach, and cecum was significantly decreased, whereas WY14643 treatment has no effect. This clearly demonstrates the protective effect of rosiglitazone against *C.albicans* mucosal colonization during type 2 diabetes.

### Rosiglitazone induced-M2a polarization is associated with the increase of anti-infectious properties

As the HFD and rosiglitazone differentially polarize the macrophage and that the responses against *Candida albicans* are different, we wondered whether the macrophage mechanisms of pathogen elimination (phagocytosis and reactive oxygen species production) were also modified.

**Figure 7:**
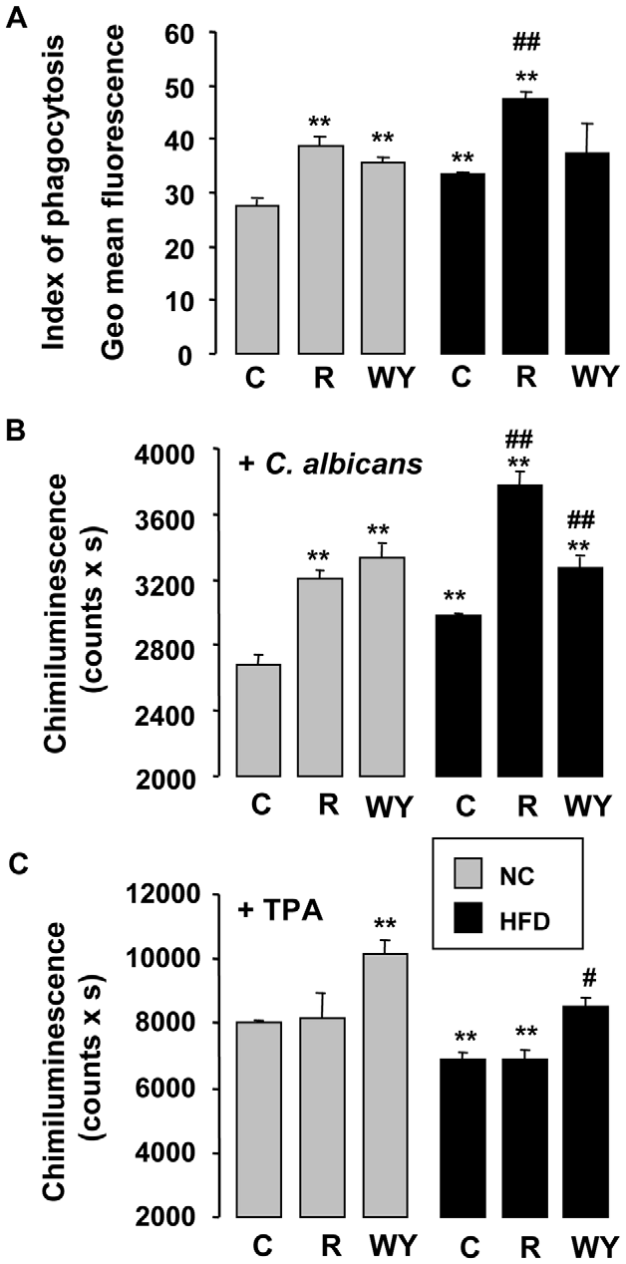
Rosiglitazone treatment promotes the candidacidal activity of macrophages from insulin resistant mice. (A) Index of phagocytosis of non-opsonised *C.albicans* by peritoneal macrophages collected from mice fed a chow (NC) or a HFD (HF) and treated or not (C) with rosiglitazone (R) or WY14643 (WY) (n = 6). The phagocytosis of non-opsonised *C.albicans* by macrophages was measured at 37°C after challenge with FITC-labeled *C.albicans* for 60 min by flow cytometry. (B–C) ROS production of peritoneal macrophages from mice fed a chow (NC) or a HFD (HF), untreated (C) or treated with rosiglitazone (R) and WY14643 (WY) (n = 6). Non-opsonised *C.albicans* (B) or TPA (C)*-*induced respiratory burst of macrophages was measured by chimiluminescence. Total chimioluminescence emission (area under the curve expressed in counts x seconds) was observed continuously for 60 min in the presence or absence of *C. albicans* or TPA. Data are represented as mean ± SEM ** p<0.01 compared to NC control (NC C); #p<0.05 compared to HF control (HF C). The data are representative of three independent experiments.

When macrophages were harvested on mice under HF diet, the number of *C. albicans* ingested increased significantly compared to macrophages from mice under chow diet (Figure 7A). Moreover, the phagocytosis is strongly increased by rosiglitazone treatment whatever the diet of the mice. Following the treatment with WY 14643, the capacity of macrophages from mice under HFD to uptake *C. albicans* is not modified.

Using chemoluminescence, we next investigated the effect of rosiglitazone or WY14643 treatments on the production of oxidizing agent by macrophage in response to *Candida* challenge (Figure 7B) or to TPA (Figure 7C). In response to a *C. albicans* challenge, the HFD induced a significant increase of ROS production. In addition, in particular rosiglitazone and to a lesser extent WY14643 amplified this production whatever the diet (Figure 7B). The TPA stimulation shows that there is no increased production of reactive oxygen species (ROS) by macrophages from mice treated with rosiglitazone under HFD (Figure 7C). However, macrophages from mice both in chow and HF fed conditions and treated with WY 14643 produce more ROS in response to TPA. The increased ROS production in macrophages from mice treated with WY14643 comes from a direct activation of NADPH oxidase.

Altogether these data show that the macrophage M2a phenotype induced by rosiglitazone is coupled with the increase of their capacity to internalize *Candida* and to produce ROS in response to *Candida* challenge.

## Discussion

Many previous studies have established the relationship between dyslipidemia, type 2 diabetes, and immune dysfunction [19]. Indeed, inflammation and inflammatory cells have been described to be key regulators in obesity and insulin resistance. Several recent studies have focused on the role of adipose tissue macrophages (ATMs) in obesity-associated inflammation and insulin resistance. In HFD-induced obesity murine model it has been demonstrated that the ATMs recruited in response to HFD express mixed M1/M2 and remodelling transcriptional profiles and that these profiles become more M2-like with extended HFD feeding [20]. In line, the human ATMs have recently been shown to express mixed M1/M2 remodelling phenotypes [4], [20], [21]. In contrast, other studies reported the recruitment of M1 pro-inflammatory polarized macrophages in adipose tissue of obese mice [1], [22]. Here, we have explored for the first time the M1/M2 polarization of macrophages of peritoneal and digestive mucosal macrophages during HFD-induced obesity. Indeed, the peritoneal macrophages, which are an abundant source of macrophages, are close to the visceral adipose tissue and hence their polarization could be influenced by the fat mass. In addition, as diabetic patients are at risk of acquiring digestive infections and as the macrophages are key cells in pathogen elimination, it is important to determinate their phenotype in intestinal tissue. In this context, we determined characteristic markers of M1 and M2 macrophage polarization and demonstrate that the peritoneal and cecal macrophages from mice under HFD exhibit an alternative M2 polarization. Indeed, the MR, Dectin-1, CD36, YM-1, YM-2 and Arginase 1 expressions are greatly enhanced by the HFD, this induction being characteristic of the M2 macrophage polarization [23]. Cytokine profiling is also an important determinant of the precise phenotype of macrophages. Peritoneal macrophages and cecal tissue from mice on HFD have a particular phenotype as they express both IL-10, characteristic of M2 phenotype, and also TNF-α, IL-6 and IL-1β proinflammatory cytokines, typical of M1 macrophages. These results are fully in line with a report of *Mantovani et al.* indicating that M2 macrophages are divided into 3 subtypes (M2a, M2b, M2c) characterized by a different cytokine profile [5]. It is clear that the peritoneal macrophages and the cell types present in the cecal tissue of insulin resistant mice correspond to M2b macrophage subtype. Our results hence confirm those of Bourlier *et al*, which demonstrated this particular M1/M2 phenotype in macrophages from adipose tissue of obese patients [4], [21] and thus reinforce the concept that metabolic abnormalities polarize macrophages, whatever their tissular origin, towards M2 macrophages.

PPARγ nuclear receptor is essential both during adipogenesis and maturation of alternatively activated M2 macrophages [2]. We demonstrate here that HFD conditions increase specifically PPARγ mRNA and protein levels. This induction of PPARγ expression by HFD is consistent with other studies, which showed the increase of PPARγ in white adipose tissue of obese mice [24] and in macrophages of monkeys under HFD conditions [25]. The induction of PPARγ is conflicting with the literature, which generally shows a decrease of PPARγ expression during inflammatory context [26], [27]. However, a pathway involved in PPARγ expression in hyperglycaemia was described. It depends on the activation of Nrf2 pathway through the generation of ROS occurring during the low grade inflammation [28], [29]. Concomitantly with this PPARγ induction, we observed an increase of MR and Dectin-1, in line with our previous results which showed that PPARγ was involved in the signalling pathway that regulates MR and Dectin-1 expression in macrophages [12], [14]. Surprisingly, in contradiction with the anti-inflammatory properties of PPARγ [30], there is also an increase of the expression of pro-inflammatory cytokines. All these data suggest that there is a lack of activation of PPARγ, caused either by a lack of endogenous ligands or by a loss of its functionality. The defect can be reversed by the addition of exogenous ligands, demonstrating that PPARγ activity is not affected by the diet. Indeed, the treatment of insulin resistant mice with rosiglitazone potentiates the induction of MR and Dectin-1 and restores the anti-inflammatory activity of PPARγ. We established that the treatment of insulin resistant mice with rosiglitazone induced a M2b to M2a switch of their peritoneal macrophages and of the cell types present in the cecal tissue, characterized by a weak production of proinflammatory cytokines (TNF-α, IL-6 and IL-1β) and IL-10 and a strong expression of the MR, Dectin-1, CD36 and TLR2. Moreover, the WY14643 treatment orientates the polarization of macrophages toward a particular phenotype, near of M2a by their cytokine profile (low production of IL-10 and TNF-α). These macrophages, however, do not represent true M2a macrophages, because they express low levels of MR and Dectin-1. The lack of induction of MR and Dectin-1 by WY14643 shows the specificity of PPARγ in the signalling pathway that regulates these two receptors. In addition, this study reinforces that PPARα may be involved with PPARγ in the suppression of pro-inflammatory cytokines. Indeed, several studies imply also an anti-inflammatory role for PPARα which interferes with the NF-kB and AP-1 inflammatory pathways [31].

The macrophage M2b phenotype induced by HFD is characterized by the increase of MR and Dectin-1-dependent microbicidal *ex vivo* functions against *C. albicans*, most common specie identified in the oral and GI mucosa of diabetic patients. However, our data underscore that insulin resistant mice are more susceptible to sustained GI *Candida* colonization than lean mice. These findings are in line with increased susceptibility to candidiasis in patients with metabolic dysregulation [9]. This discrepancy between the *ex vivo* and *in vivo* data is attributed to the fact that the *in vivo Candida* elimination involves both opsonin-dependent and independent host defence mechanisms, while in our *ex vivo* experimental conditions, only the elimination of non-opsonized *C. albicans* is implicated. Altogether, these results strongly suggest that the HFD may affect other immune functions involved in the opsonized *C. albicans* elimination through M1 activation. Consistent with this hypothesis, we demonstrated that the CD11b complement receptor type 3, the principal adhesion receptor on leukocytes for *Candida albicans* [32] is strongly decreased in macrophages from mice under HF diet, suggesting a default of pathogen-opsonised recognition in this dyslipidemic context. In line, different disturbances in innate humoral immunity were previously described in diabetic patients. Indeed, the expression of complement receptor 3 on monocytes from non-insulin-dependent diabetes patients was strongly decreased and the CD55 and CD59 complement regulatory protein-positive monocytes were lower in type 2 diabetic patients [33]–[36].

Interestingly, the rosiglitazone-induced M2a polarization compensates the defect of the humoral innate immune response. Indeed, this M2a macrophage polarization is correlated with improved elimination of GI *C. albicans*. In this state the peritoneal macrophages strongly express the MR and Dectin-1 on their surface and thus the antifungal functions associated with these receptors (phagocytosis and ROS production) are robustly increased. Moreover, the mucosal macrophages present on the infection site share the same M2a phenotype and hence could participate actively to *Candida* elimination in digestive tract. These data are consistent with previous results showing that PPARγ specific ligands enhance fungal clearance in immunodeficient Rag-2-/- mice, reinforcing the concept that the M2a polarization induced by PPARγ ligands is able to thwart the lack of humoral immune response [14]. Moreover, our data indicate that short-term rosiglitazone treatment was ineffective to decrease blood glucose levels. These observations are consistent with previous studies showing that 3 weeks of rosiglitazone treatment are not sufficient to reduce hyperglycemia in a transgenic mouse model of type 2 diabetes and that in general 4–5 weeks of treatment are necessary to reduce blood glucose level in mice on HFD [37], [38]. Likewise, in humans, at least 2 weeks of therapy are necessary to see an effect on blood glucose level. Altogether these data reveal that the anti-fungal effect of rosiglitazone is independent of changes in blood glucose level and is strictly correlated to the M2a macrophage polarization.

On the other hand, the decrease in *Candida* GI colonization is not observed in mice treated with the WY14643 (PPARα ligand), demonstrating the specific effect of PPARγ. Unlike rosiglitazone, WY14643 increase slightly MR and Dectin-1 and thus the antifungal functions of macrophages are not induced. In addition, the absence of a WY14643-mediated induction of the expression of TLR2, receptor known to collaborate with Dectin-1 to promote macrophage antifungal functions [39], contributes to the lack of induction of anti-infectious functions of macrophages and hence explains the ineffectiveness of PPARα ligands in *Candida* elimination.

In conclusion, we established that the peritoneal macrophages and the cell types present in the cecal tissue during metabolic disorders present a M2b phenotype. This polarization under insulin resistance is associated with an increased susceptibility to GI fungal infections. Rosiglitazone treatment induces a phenotypic M2b-to-M2a switch of macrophages, which promote mucosal defence mechanisms against GI candidiasis. Our findings hence demonstrate a dual benefit of PPARγ ligands, which are able to promote mucosal defence mechanisms against GI candidiasis on the one hand through M2a polarization and on the other hand by lowering blood glucose levels.

## Materials and Methods

### Mice and experiments

All animal experimentation was conducted in accordance with accepted standards of humane animal care. Mouse experiments were approved and performed according to the guidelines of the Toulouse University Animal Safety Committee and of the Regional Safety Committee (Procedure 1992). This study was carried out in accordance with Approval No. A3155503 and all procedures for animal care and maintenance conformed with the French and European Regulations (Law 87-848 dated 19/10/1987 modified by Decree 2001-464 and Decree 2001-131 relative to European Convention, EEC Directive 86/609 dated 24/11/1986).

The model of type 2 diabetes was induced on 6-wk-old male wild-type C57BL/6 mice (Janvier, France) by a high fat diet (Safe, 49.5% of calories from lipids) for 12 weeks. The mice's weight was monitored once a week. All cages were changed twice weekly, and all manipulations of the animals were done in a laminal blow hood under aseptic conditions. The photoperiod was adjusted to 12 h light and 12 h dark. After 12 weeks of the high fat diet, we established esophageal and GI candidiasis on half of the mice by performing intraesophageal infection with 5×107 viable cells of *Candida albicans* in sterile saline solution (500 µl/mouse). At this level of infection, the fungal colonization in mice under chow diet was undetectable after one week, as described previously [15]. On day 8 after infection, all mice were euthanized using CO2 asphyxia.

### Treatment groups

Therapeutic studies were performed on separate groups of six mice, infected or not by *C. albicans*. Mice were treated with rosiglitazone (Cayman Chemical) or WY 14643 (Calbiochem) 1 day before, the day of the infection with *C. albicans* and then every 2 days (28 µg/10 g of mouse) (five injections). Control groups received saline solution only with DMSO. The body weight of each mouse was recorded daily.

### IntraPeritoneal Glucose Tolerance Test (IPGTT)

After 12 weeks of the HF diet, 3 animals per group were fasted for approximately 6 hours and a solution of 20% of glucose was then administered by intra-peritoneal (IP) injection (1 g of glucose/kg mouse) and blood glucose was measured at different time points (0, 15, 30, 45, 120 min).

### IntraPeritoneal Insulin Sensitivity Test (IPIST)

After 12 weeks of the HF diet, 3 animals per group were fasted for approximately 6 hours and insulin was then administered by intra-peritoneal injection (0.5 U/kg of mouse) and blood glucose was measured at different time points (0, 15, 30, 45, 60 min).

### *Candida albicans* strain

The strain of *Candida albicans* used throughout these experiments was isolated from a blood culture of a Toulouse-Rangueil Hospital patient. The isolate was identified as *C. albicans* based on common laboratory criteria (BichroLatex *C.albicans®* (Fumouze), Mini API® test (Bio Merieux)) and cultured on Sabouraud dextrose agar (SDA) plates containing gentamicin and chloramphenicol. *C. albicans* was maintained by transfers on SDA plates. Growth from an 18 to 24 h SDA culture of *C. albicans* was suspended in sterile saline.

Fluorescent *C.albicans* was prepared by adding *C.albicans* to fluoroscein isothiocyanate (FITC, Sigma) dissolved in sodium carbonate buffer (pH 9.5) at room temperature for 3 h and washed by centrifugation three times in sodium carbonate buffer before storage in aliquots of water at 4°C.

### Quantification of *C. albicans* in the esophagus and GI tract

*Cell lysis and DNA extraction*. After mouse euthanasia, esophagus, stomach and cecum were aseptically removed to evaluate *C. albicans* colonization. These organs were then crushed using lysing matrix tubes (MP Biomedicals). A total of 250 µl of each tissue sample homogenate was resuspended in 200 µl of lysis buffer for 2 h at 65°C (High Pure PCR Template preparation kit, Roche diagnostics). Another lysis step was then performed with binding buffer for 10 minutes at 72°C. DNA was then extracted with isopropanol. The lysis reaction was stopped with inhibitor buffer and a serie of 2 washes were then performed. DNA was eluted with an elution buffer.

*Light Cycler-based PCR assay*. The Light Cycler PCR and detection system (Roche Diagnostics) was used for amplification and online quantification. PCR analysis was performed as described [14]. Serially diluted samples of genomic fungal DNA obtained from *C. albicans* cultures (40.106 cells) were used as external standards in each run. Cycle numbers of the logarithmic linear phase were plotted against the logarithm of the concentration of template DNA to evaluate the number of yeast cells present in each tissue sample homogenate.

### Preparation of mouse resident peritoneal macrophages

After euthanasia, resident peritoneal cells were harvested by washing the peritoneal cavity with 5 ml of sterile PBS medium. Collected cells were centrifuged at 400 g for 10 min and the cell pellet was suspended in Dulbecco's modified Eagle's medium (DMEM, Gibco Invitrogen Corporation) supplemented with glutamine (Gibco Invitrogen Corporation), penicillin, streptomycin (Gibco Invitrogen Corporation) and 5% heat-inactivated fetal calf serum. Cells were allowed to adhere for 2 h at 37°C and 5% CO2. Non adherent cells were then removed by washing with PBS.

### Flow cytometry

The analysis was performed on non adherent macrophages [15]. Surface expressed Dectin-1 or CD36 was detected respectively using FITC-Dectin-1 mAb (Serotec) or PE-CD36 mAb (Santa Cruz) and was compared with an irrelevant appropriate isotype control. The labeled mAbs anti-CD11b-Alexa 647, and anti-TLR2-Alexa 488 were obtained from Serotec. To evaluate the Mannose Receptor (MR) surface expression, we have used MR-specific ligand conjugated with FITC (Sigma). A population of 10 000 cells was analyzed for each data point. All analyses were done in a Becton Dickinson FACScan using CellQuestPro software.

### Reverse transcription and real-time PCR

Total RNA obtained from peritoneal macrophages or cecum tissue was prepared with RNeasy® Mini Kit columns (Qiagen) using the manufacturer's protocols. Synthesis of cDNA was performed from 1 µg of total RNA with QuantiTect® Reverse Transcription (Qiagen) according to the manufacturer's recommendations and primed with hexamers. Quantitative real-time PCR was performed on a LightCycler system (Roche Diagnostics) using QuantiFastTM SYBR® Green PCR (Qiagen). Ten microliters of reaction mixture was incubated; the amplifications were performed for 50 cycles (10 s at 95°C and 60 s at 60°C). The primers (at a final concentration of 10 mM) were designed with the software Primer 3 and listed in Table 1. β-actin mRNA was used as the invariant control.

**Table 1:** Primers sequences used in quantitative PCR experiments.

*Gene*	sequence
*β-actin*	sense 5′AGC CAT GTA CGT AGC CAT CC3′antisense 5′CTC TCA GCT GTG GTG GTG AA3′
*YM-1*	sense 5′AAT GAT TCC TGC TCC TGT GG3′antisense 5′ACT TTG ATG GCC TCA ACC TG3′
*YM-2*	sense 5′CAC GGC ACC TCC TAA ATT GT3′antisense 5′GCT GGA CCA CCA GGA AAG TA3′
*TNF-α*	sense 5′AGG CTG TGC ATT GCA CCT CA3′antisense 5′GGG ACA GTG ACC TGC ACT GT3′
*IL-10*	sense 5′TTT TCA CAG GGG AGA AAT CG3′antisense 5′CCA AGC CTT ATC GGA AAT GA3′
*IL-1β*	sense 5′GAT CCA CAC TCT CCA GCT GCA3′antisense 5′CAA CCA ACA AGT GAT ATT CTC CAT G3′
*IL-6*	sense 5′AAG TGC ATC ATC GTT GTT CAT ACA3′antisense 5′GAG GAT ACC ACT CCC AAC AGA CC3′
*Arginase1*	sense 5′AGA GCT GAC AGC AAC CCT GT3′antisense 5′GGA TCC AGA AGG TGA TGG AA3′
*PPARγ*	sense 5′AAT CCT TGG CCC TCT GAG AT3′antisense 5′TTT TCA AGG GTG CCA GTT TC3′
*PPARα*	sense 5′GGT CAC CTA CGA GTG GCA TT3′antisense 5′GAG GGT TGA GCT CAG TCA GG3′
*PPARβ/δ*	sense 5′GTA CTG GCT GTC AGG GTG GT3′antisense 5′TGG AGC TCG ATG ACA GTG AC3′
*CD36*	sense 5′GCA GAA TCA AGG GAG AGC AC3′antisense 5′GAG CAA CTG GTG GAT GGT TT3′
*Mannose Receptor*	sense 5′ATG CCA AGT GGG AAA ATC TG3′antisense 5′TGT AGC AGT GGC CTG CAT AG3′
*CD11b*	sense 5′AGA TCG TCT TGG CAG ATG CT3′antisense 5′GAC TCA GTG AGC CCC ATC AT3′
*Dectin-1*	sense 5′5′CAT CGT CTC ACC GTA TTA ATG CAT3′, antisense 5′CCC AGA ACC ATG GCC CTT3′

### ELISA Cytokine titration

Peritoneal macrophages were added to 96 well plates (2.105macrophages/well) then stimulated with non-opsonised *C.albicans* at a yeast-to-macrophage ratio of 3∶1 for 1 h. Supernatants were recovered and frozen at −70°C before analysis. The production of TNF-α and IL-10 in the cell supernatants was determined with a commercially available OptiEIA kit (BD Biosciences) according to the manufacturer's instructions.

### Western Blot Analysis

Total protein lysates were extracted with 25 mM Tris pH 8, 200 mM glycine, 0.25% SDS and anti-protease cocktail (Roche). After protein transfer,membranes were incubated overnight at 4°C with a rabbit polyclonal anti-PPARγ (Santa Cruz) and then for 1 h at 21°C with a peroxidase conjugate secondary antibody. Membranes were washed, and proteins were visualized with the SuperSignal West Pico Chemiluminescent Substrate (ThermoScientific).

### Phagocytosis assay

For analysis of phagocytosis of *C.albicans*, cultured macrophages were challenged with six FITC labeled yeasts per macrophage and phagocytosis was initiated at 37°C in an atmosphere of 5% CO2 in 48 well Falcon plates (5.105 macrophages/well). Phagocytosis was stopped after 60 min by washing the macrophages with ice-cold PBS. Macrophage monolayers were incubed with ice-cold PBS-EDTA (5 mM) and gently scarped. The number of *C.albicans* engulfed by macrophages was determined using FACS based approach. The distinction between internalised yeast cells and yeasts attached to macrophages surface was done *via* quenching of FITC fluorescence by trypan blue. The remaining fluorescence was quantified on a Becton Dickinson FACScan using CellQuestPro software and used as indicator of the phagocytosis efficiency.

### Assay for oxidizing agent production

The macrophages were plated in 96 well Falcon plates (3.105 macrophages/well). The oxygen dependent respiratory burst of macrophages was measured by chemiluminescence in the presence of 5-amino-2,3-dihydro-1,4-phthalazinedione (luminol) using a thermostatically (37°C) controlled luminometer (Wallac 1420 Victor2). The luminol detects both reactive oxygen and nitrogen intermediates (O2.-, ONOO-, OH.). The generation of chemoluminescence was monitored continuously for 1 h after incubation of the cells with luminol (66 µM) and after 12-O-tetradecanoylphorbol-13-acetate (TPA, 100 µM) or after *C. albicans* challenge at a yeast-to-macrophage ratio of 3∶1. Statistical analysis was performed using the area under the curve expressed in counts x seconds.

### Immunohistochemical analysis of cecum macrophages

Cecum tissues of wild-type mice treated or not with rosiglitazone or WY 14643 were removed, and carefully opened longitudinally [14].After washing in PBS, the tissues were fixed for 20 min in PBS containing 4% paraformaldehyde. Then, fixation was quenched with 100 mM glycine (pH 7.4) and samples were permeabilized for 10 min in Triton X-100 and washed in PBS. Fixed tissues were blocked with solution 1 (75 mM NaCl, 18 mM Na3 citrate, 2% goat serum, 1% BSA, 0.05% Triton X-100, and 0.02% NaN3) for 2 h at room temperature. The tissues were stained with F4/80, MR and Dectin-1 primary Antibodies (goat anti-mouse, Santa-cruz for F4/80; goat anti-rabbit, Santa-cruz for MR; goat anti-rat, Acris antibodies for Dectin-1) diluted (1/500) in solution 1 for 48 h at 4°C. Then, tissues were washed in solution 2 (75 mM NaCl, 18 mM sodium citrate, and 0.05% Triton X-100) for 1 h30 and rinsed in PBS. The tissues were then incubated for 1 h at room temperature with Alexa conjugated secondary antibodies (Alexa 647, 488, 546 for F4/80, MR and Dectin-1 respectively, Molecular Probes) diluted in solution 1. Finally, they were washed in solution 1 for 30 min at room temperature. All preparations were mounted with Kaiser's glycerol gelatin (Mercks). All microscopy imagery was performed with a Zeiss LSM 510 (ConfoCor, Zeiss).

### Statistical analysis

For each experiment, the data were subjected to one-way analysis of variance followed by the means multiple comparison method of Bonferroni-Dunnett. p<0.05 was considered as the level of statistical significance.
